# Micro-Mechanism of Uniaxial Compression Damage of Layered Cemented Backfill in Underground Mine

**DOI:** 10.3390/ma15144846

**Published:** 2022-07-12

**Authors:** Xinming Chen, Haowen Zhang, Yuping Wu, Huazhe Jiao, Liuhua Yang, Qinting Wang, Wenxiang Zhang

**Affiliations:** 1 School of Civil Engineering, Henan Polytechnic University, Jiaozuo 454000, China; chenxinming@163.com (X.C.); 212008020007@home.hpu.edu.cn (H.Z.); yanglh@hpu.edu.cn (L.Y.); wangqt@hpu.edu.cn (Q.W.); 212008020006@home.hpu.edu.cn (W.Z.); 2 Collaborative Innovation Center of Coal Work Safety and Clean High Efficiency Utilization, Jiaozuo 454000, China; 3 Energy Economics Research Center, Henan Polytechnic University, Jiaozuo 454000, China

**Keywords:** layered cemented filling, constitutive damage model, PFC2D, energy conversion, failure mode

## Abstract

Layering of filling body is common in large-scale filling areas. In this paper, the cement–sand ratio of 1:8 is based on the configuration of 68%, 70%, 72% and 74%; four concentrations; and filling layers of one-, two-, three- and four-layered cemented filling samples. Combined with a uniaxial compression test and two-dimensional particle flow software (PFC2D), the mechanical properties and failure modes were explored. The results show that the concentration can strengthen the uniaxial compressive strength of the filling body while increasing the filling times weakens its power; therefore, the constitutive damage model was constructed. It was found that the initial layered damage existed in the layered filling, and the total damage showed an upward trend of first fast and then slow; the main failure modes of one-layer and two-layer backfills showed prominent shear failure characteristics, and the three-layer and four-layer fillings were closer to tensile failure. From the crack generation to the specimen failure, there is a mutual conversion between different energies.

## 1. Introduction

Mining is one of the essential conditions to ensure the rapid development of China’s economy, but mining also brings problems such as goaf collapse and mining waste pollution [1,2,3,4,5,6,7,8,9,10,11,12,13,14]. The filling mining method can effectively control stope ground pressure and stope deformation while making full use of mine solid waste tailings, which not only ensures the safety of mining but also solves the problem of environmental pollution to a certain extent [15,16,17,18,19,20,21,22,23,24,25,26], so it is extensive use of various metal and non-metal mines.

Tailings cemented backfill is a multiphase composite material formed by mixing tailings, cement and water according to a specific ratio. As an essential part of the filling mining method, many scholars conducted studies on its physical properties and failure mode. Cao S et al. [27] found that the dynamic compressive strength of cemented tailings composites increased exponentially with the increase in average strain rate, and the pore size of cemented tailings composites increased with the increased average strain rate. The fractal dimension of the pore area increased linearly with an increased average strain rate. Gai X et al. [28] improved the uniaxial compressive and splitting tensile strength by adding fibers into the tailings cement. The addition of fibers transformed the failure mode of the tailings cement into ductile failure. Wang et al. [29] found that all the damage curves of tailings cemented backfill showed S-shaped distribution by establishing the constitutive model of tailings cemented backfill. The first slowly increased, then rapidly increased and finally slowly rose to the highest point.

The above studies mainly focused on the complete filling body, and with the consumption of external mineral resources, deep mining has gradually become the mainstream mining trend [30,31]. The development of deep mining technology is accompanied by the rapid increase in the number and size of goaf [32,33,34,35,36,37]. Limited by the current domestic filling technology, it is almost impossible to fill large-scale mined-out areas once, which leads to the stratification of the filling body due to layer-by-layer filling [38,39,40,41]. Different from the complete backfill, the structural characteristics and mechanical properties of layered backfill are more complicated. In order to study the mechanical properties and failure mode of the layered filling different from the complete filling body, this paper focused on the layered filling body samples with the cement–sand ratio of 1:8. The mechanical properties of the layered filling under different concentrations and different layered states were measured by the uniaxial compression test, and the constitutive damage model of the filling body was established to explore the damage evolution characteristics. At the same time, the particle flow software PFC2D was used to simulate and analyze the micro failure mechanism from three aspects: fracture evolution law, failure mode and energy conversion.

## 2. Materials and Methods

### 2.1. Material

**Figure 1:**
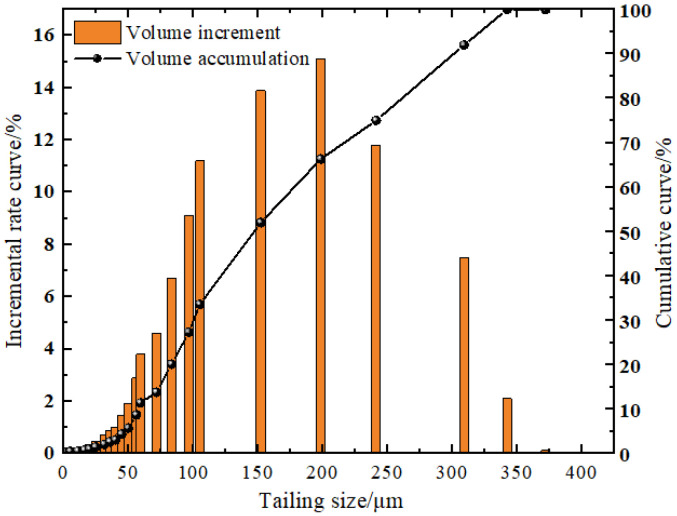
Particle size distribution curve of tailings.

The Portland cement used in the test was PC.42.5 cement produced by a cement plant. The whole tailings material was taken from a metal mine in Shandong. Its chemical composition is shown in Table 1. After drying tailings, the particle size of tailings was analyzed by a laser particle size analyzer, as shown in Figure 1.

It can be seen from the figure that the particle size of the tailings is mainly distributed between 0.6 μm and 350 μm, and the particles with a particle size smaller than 125.32 μm account for more than 50%. The average particle size is 141.32 μm.

### 2.2. Methods

#### 2.2.1. Sample Preparation

**Figure 2:**
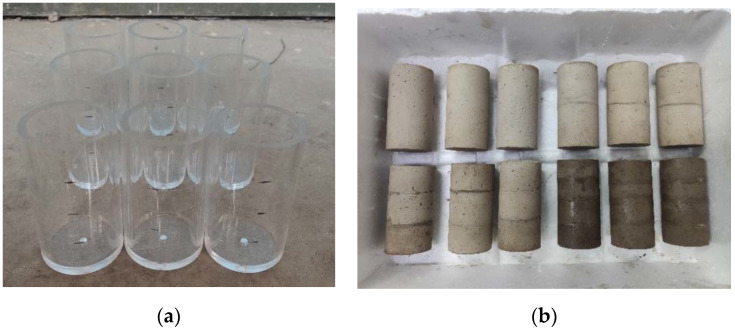
Filling Mold and Filling Body Sample; (**a**) Filling Mold, (**b**) Filling Body Sample.

Silicate cement and early dried tailings were configured according to the cement–sand ratio of 1:8 and slurry concentration of 68%, 70%, 72% and 74%. The sample mold adopts a cylinder high transparent acrylic tube with a height of 100 mm and an inner diameter of 50 mm. This paper mainly studied the influence of filling layers on the strength of the filling body. Before production, lubricating oil was smeared inside the mold, and the slurry, after uniform stirring, was poured into the mold. The filling time interval of each layer was 24 h. After the filling of each layer was completed, it was left to stand for 24 h, then de-molded and placed in a standard curing environment for 28 d. A uniaxial compression test was carried out after the maintenance. The filling mold and partial filling body samples are shown in Figure 2.

#### 2.2.2. Numerical Simulation

**Figure 3:**
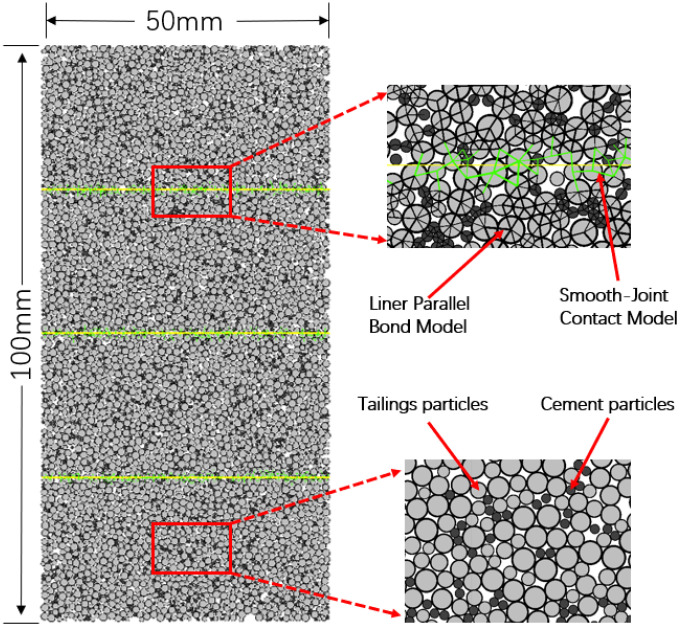
Numerical model of four-layer horizontal filling body.

In order to analyze the failure mode of layered backfill from the microscopic point of view better, this paper used PFC2D to establish a discrete element model of layered backfill. It takes 74% slurry concentration backfill as an example to simulate the uniaxial compression process of different layers of backfill. In order to be as close as possible to the actual situation, a numerical calculation model was established as a rectangle with a length of 100 mm and a height of 50 mm. Two particle models were used to simulate the tailings and cement particles, and the radius of cement particles was slightly smaller than that of tailings particles. The tailings model with a specific gradation was filled in the rectangle according to a specific porosity. Then a certain number of cement particles were randomly generated between the tailing particles. In order to make the force of the model closer to the actual strength of the filling body sample, the parallel bond model was adopted between the particles [18,42]. The four-layer horizontal filling body was taken as an example to establish its numerical model, which is shown in Figure 3, through the bond transfer force and moment between particles and smooth bonding between layers.

As shown in Figure 3, light color particles represent tailings particles, dark color particles represent cement particles, green lines represent smooth joint contact between particles and black lines represent a parallel bonding model between particles. Without any boundary conditions on both sides of the model, the uniaxial compression test was simulated by applying the downward and upward motion rates to the top and bottom walls, and stress–strain, energy and number of cracks during loading were recorded.

## 3. Results and Discussion

### 3.1. Analysis of Mechanical Properties of Layered Filling Body

#### 3.1.1. Uniaxial Compressive Strength of Layered Backfill

**Figure 4:**
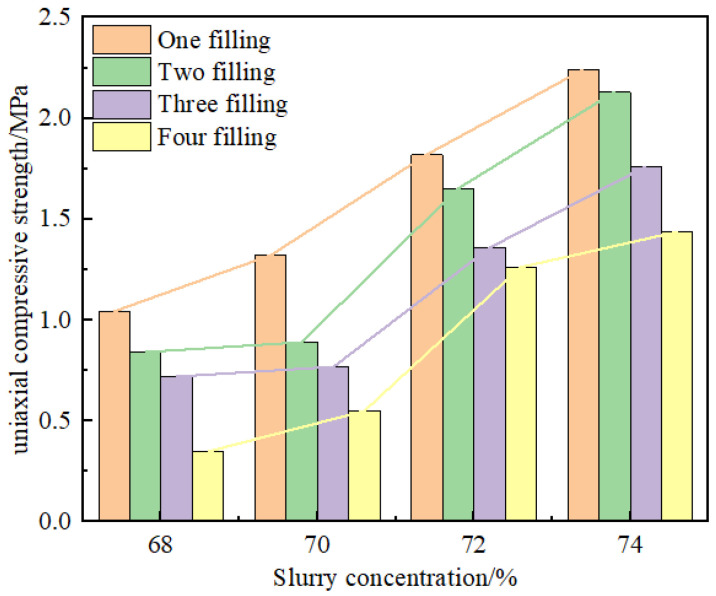
Histogram of compressive strength.

The uniaxial compressive capacity of the filling body is one of the most intuitive standards to test the quality of a filling body. The slurry concentration and filling layer number are essential factors affecting the strength of the filling body. This experiment adopted the LGS5000 press produced by the precise instruments of the Kunshan egret industry. Each group of samples with the average value of the compressive results of the five samples as the test results. If one of the test data of five samples exceeded the average value by 15%, the data would be rejected, and the average value of the remaining data was taken as the test result; the test results are shown in Table 2. Figure 4 is the histogram of compressive strength distribution, from which we can intuitively see the quantitative relationship between the uniaxial compressive strength of the filling body and different filling times or concentrations. The influence degree of slurry concentration and filling times on the uniaxial compressive strength of the filling body was analyzed by two-factor variance, and the results are shown in Table 3, Table 4 and Table 5.

It can be seen from Table 3 that the *p* values of slurry concentration and filling times are all less than 0.05, which indicates that slurry concentration and filling times have significant effects on the uniaxial compressive strength of the filling body and compared with filling times, the F value of slurry concentration is larger, which proves that slurry concentration has more significant effects on the uniaxial compressive strength of filling body. At the same time, the S-N-K method was used to compare the slurry concentration and filling times at different levels. It was found that slurry concentration was positively correlated with uniaxial compressive strength while filling times were negatively correlated with uniaxial compressive strength. When the slurry concentration is 74% and the filling times are 1, the uniaxial compressive strength of the filling body is the highest.

#### 3.1.2. Elastic Modulus of Layered Filling Body

Elastic modulus measures an object’s ability to resist elastic deformation. The elastic modulus of the layered filling body is an important parameter affecting its performance. High elastic modulus means that deformation requires high stress. The calculation formula of elastic modulus E is shown in Equation (1).

(1) $$E=\frac{\sigma_{0.8}-\sigma_{0.2}}{\varepsilon_{0.8}-\varepsilon_{0.2}}$$

where $\sigma_{0.8}$ and $\sigma_{0.2}$ are the 80% and 20% peak stress, respectively; $\varepsilon_{0.8}$ and $\varepsilon_{0.2}$ are the strain corresponding to 80% and 20% peak stress, respectively.

By taking 74% slurry concentration as an example, the elastic modulus of cemented tailings backfill with different layers was calculated according to Equation (1) as shown in Table 6.

### 3.2. Damage Constitutive Equation and Damage Evolution of Layered Cemented Filling Body

#### 3.2.1. Establishment of Damage Constitutive Equation

In the layered filling process, due to different hydration times and other external factors, the filling body on the surface and under the layered structure is prone to damage such as pores and cracks, which is defined as the initial layered damage. With the increase in filling times, the internal initial delamination damage gradually accumulates, thereby reducing the mechanical properties of the filling body. Therefore, it can not be limited considering the influence of external loads on the mechanical properties of the filling body, and a constitutive damage equation considering structural characteristics should be established.

The initial delamination damage $D_{n}$ is defined according to the macroscopic damage mechanics available in Equation (2).

(2) $$D_{n}=1-\frac{E_{n}}{E_{1}}$$

where $E_{n}$ is the elastic modulus of the filling body with n layers; $E_{1}$ is the elastic modulus of the complete filling body.

Under the external load, some randomly distributed micro defects gradually occur inside the filling body. This damage is defined as the loading damage of the filling body. According to the Lemaitre strain equivalence principle, the loading damage constitutive equation of the filling body can be defined as Equation (3).

(3) $$\sigma =E\left(1-D_{s}\right)\varepsilon$$

where $D_{s}$ is the damage of filling body under load; E is the elastic modulus of the filling body without damage; ε for filling body strain;

Based on the strain equivalent assumption of the filling body, when the filling body is loaded to the strain ε level, the loading damage can be expressed as Equation (4).

(4) $$D_{s}=1-e^{-{\left(\frac{\varepsilon }{\varepsilon_{0}}\right)}^{m}}$$

where m and $\varepsilon_{0}$ are parameters for the damage constitutive model;

Different from the complete backfill, in the layered backfill, the damage of the backfill under load is composed of two parts, one part is the initial delamination damage, and the other part is the load damage. Under the action of layered structure and load, the two kinds of damage are coupled and influence each other. Therefore, the stress–strain relationship of the layered filling body under the condition of internal damage is Equation (5).

(5) $$\sigma =E_{n}\left(1-D_{s}\right)\varepsilon$$

Substituting Equation (2) into Equation (5), the stress–strain relationship expressed by the initial delamination damage and the load damage can be obtained, as shown in Equation (6).

(6) $$\sigma =E_{0}\left(1-D_{s}\right)\left(1-D_{n}\right)\varepsilon$$

From Equation (6), the total damage D of the layered backfill after two kinds of damage coupling under load is:

(7) $$D=D_{n}+D_{s}-D_{n}D_{s}$$

Equations (2) and (4) are substituted into Equation (7):

(8) $$D=1-\frac{E_{n}}{E_{0}}\exp\left[-{\left(\frac{\varepsilon }{\varepsilon_{0}}\right)}^{m}\right]$$

From Equations (3) and (8), the axial stress $\sigma_{1}$ under the constitutive damage model of layered backfill under the uniaxial compression test is shown in Equation (9).

(9) $$\sigma_{1}=E_{0}\left(1-D\right)\varepsilon_{1}$$

where $\sigma_{1}$ is the axial stress for filling body; $\varepsilon_{1}$ is the axial strain of filling body.

Equation (8) can be replaced by Equation (9):

(10) $$\sigma_{1}=E_{n}\exp\left[-{\left(\frac{\varepsilon }{\varepsilon_{0}}\right)}^{m}\right]\varepsilon_{1}$$

Pairwise Equation (10) Derivation:

(11) $$\frac{\partial \sigma_{1}}{\partial \varepsilon_{1}}=E_{n}\exp\left[-{\left(\frac{\varepsilon }{\varepsilon_{0}}\right)}^{m}\right]\left[1-\frac{\varepsilon_{1}m}{\varepsilon_{0}}{\left(\frac{\varepsilon }{\varepsilon_{0}}\right)}^{m-1}\right]$$

Assuming that when the filling body reaches the peak value in the stress–strain curve, $\sigma_{1}=\sigma_{f}$, $\varepsilon_{1}=\varepsilon_{f}$ and then $\frac{\partial \sigma_{f}}{\partial \varepsilon_{f}}=0$, Equations (10) and (11) can simultaneously be obtained:

(12) $$m=\ln\left(\frac{\sigma_{f}}{E_{n}\varepsilon_{f}}\right)$$

(13) $$\varepsilon_{0}=\varepsilon_{f}{\left(m\right)}^{1/m}$$

#### 3.2.2. Analysis of Damage Evolution Characteristics

**Figure 5:**
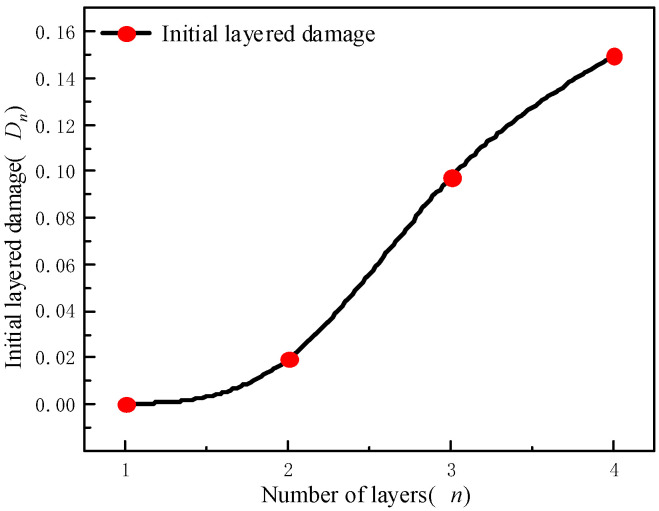
Relationship curve between delamination number and initial delamination damage.

According to Table 6 and Formula 2, the relationship between the initial damage and the number of filling layers can be obtained, as shown in Figure 5.

Figure 5 shows that the initial delamination damage of the filling body increases with the number of layers. When the number of layers increases from one layer to four layers, the initial delamination damage of the layered filling body increases by 1.95%, 9.74% and 14.94%, respectively, which also proves the coupling effect of layers aggravate the initial damage of the filling body.

The damage constitutive model parameters of different layered fillings can be obtained by substituting Table 3 data into Equations (12) and (13), as shown in Table 7.

**Figure 6:**
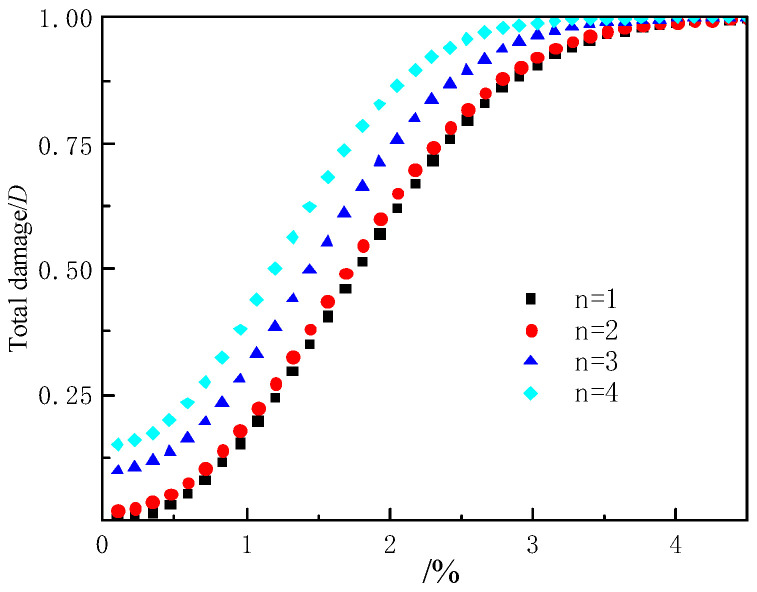
Total damage evolution curve of layered backfill.

According to Equation (8) combined with Table 4 data, the total damage evolution curve of filling body under delamination and load coupling can be obtained, as shown in Figure 6.

It can be seen from Figure 6 that for layered backfill, there is initial damage before its strain occurs. Since the first layer of backfill is completely filled, there is no initial damage, so the initial total damage is 0. The second layers of backfill begin to show initial damage, but the damage increase is not apparent. When the filling body layers are the third and fourth layers, the initial damage increases significantly. With the increase in strain, the total damage of backfills with different layers increases sharply at first, then slowly and finally gradually tends to the growth mode of 1. The total damage growth rate of fillings with different layers is roughly the same. When the damage to the filling body is the same, the more layers of the filling body are, the greater the strain is. This is because when the layered filling body is under load, the layered structure surface closes under load, resulting in strain increases.

### 3.3. Micro-Failure Mode Analysis of Layered Cemented Backfill

#### 3.3.1. Failure Mode Analysis of Layered Cemented Filling Body

**Figure 7:**
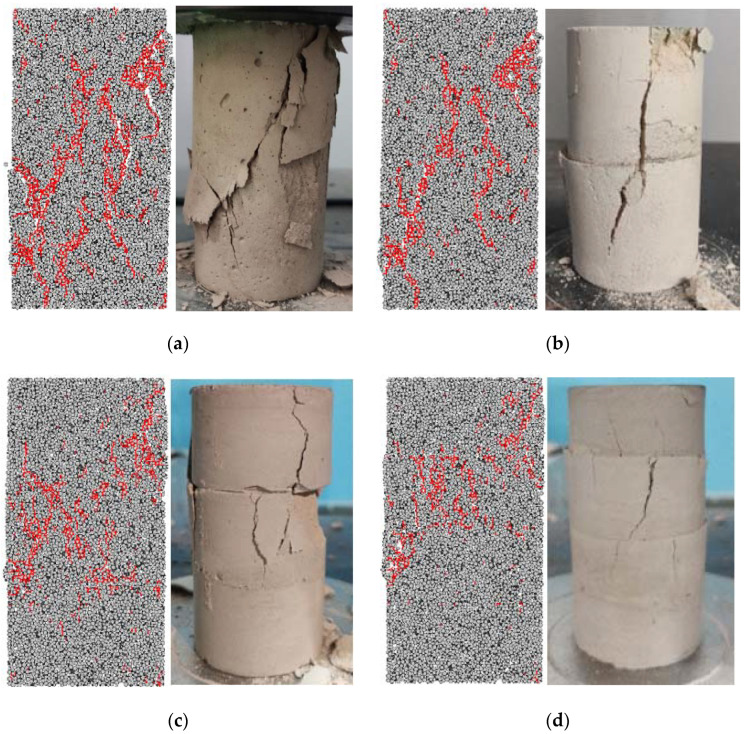
Failure mode of numerical model of horizontally layered filling body; (**a**) one layer, (**b**) two layers, (**c**) three layers, (**d**) four layers.

Figure 7 shows the numerical failure mode and the actual failure mode of different layered filling samples. It can be seen from the figure that the failure mode simulated by PFC2D is basically consistent with the virtual failure mode. The main failure modes of the first filling sample and the second filling sample both showed obvious shear failure characteristics. The sample appeared to be a shear plane composed of multiple oblique cracks with an angle of 45° to the concentrated load direction, which ran through the whole sample and led to the failure of the sample. Compared with the one-layer backfill sample, the angle between the shear plane and the concentrated load direction of the two-layers backfill sample is smaller, and the failure mode gradually evolves to the tensile failure throughout the whole sub-layer. The main failure modes of three layers backfill sample and four layers backfill sample are closer to tensile failure, and the failure areas are mainly concentrated in the top and middle layers. At the same time, after the uniaxial compression test of the backfill sample was filled multiple times, the sub-layers had different degrees of dislocation phenomenon. There are some defects, such as pores and cracks between the layers, and the surface of the layered layers is uneven. In addition, the filling time difference forms a low-strength interlayer between adjacent layers. During the loading test, layered defects and low-strength interlayers reduce the overall compressive strength of the filling body, making it more prone to failure, which leads to the gradual decrease in the number of tiny cracks in the filling body during the failure process with the increase in layers of the filling body. From the damage evolution curve, it can be seen that before the external load is applied, the layered filling body itself has initial damage. When the load is applied, due to the closure of the layered structure surface, the damage first occurs in the filling body. With the increase in the load, the damage gradually accumulates and gradually expands to the periphery. When the number of layers is more, the damage accumulation of the filling body is faster, which leads to the failure of the filling body with more layers, such as the filling body with three and four layers, due to the excessive accumulation of damage in the middle layer, so the load cannot be continuously transferred to the bottom, so the bottom is relatively complete and maintains a certain strength.

#### 3.3.2. Analysis of Fracture Evolution Law of Layered Cemented Filling Body

**Figure 8:**
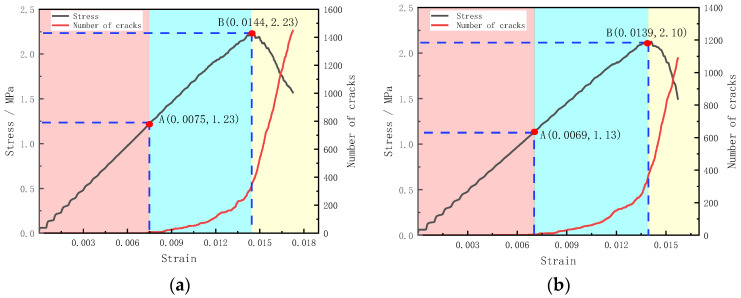

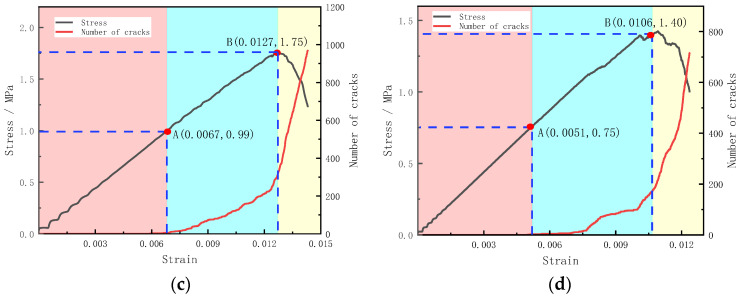
Composite diagram of stress–strain curve and crack increment curve of numerical model of horizontal layered filling body; (**a**) one layer, (**b**) two layers, (**c**) three layers, (**d**) four layers.

The composite diagram of the stress–strain curve and crack increment curve for the numerical model of the horizontal layered filling body is shown in Figure 8. Table 8 shows the relationship between stress–strain and crack increment, which can more intuitively reflect the stress and strain values when the crack occurs and the crack growth rate changes.

In the PFC2D simulation, the connection between particles was realized by parallel bonding bonds, which can establish an elastic interaction between two particles close to each other, to transfer the force and moment between particles. However, when the force acting on the bonding bond is greater than its maximum bearing limit, the bonding bond fails, and the two particles become independent units that cannot transmit force. It can be seen from Figure 6 that in the early stage of loading, the bond strength between particles in the filling body is greater than the contact force between particles, so the early loading force cannot destroy the bond between particles, and there are no cracks in the filling body. As the loading force increases, the contact force between particles increases. When the stress of the filling body reaches about 55% of the peak stress, the contact force between some particles begins to exceed the bond strength, which leads to the fracture of the bond. At this time, the cracks begin to appear and gradually develop. When the loading enters the later stage, more and more particles break the bond, and the number of cracks gradually increases. When the filling body stress reaches 90–100% of the peak stress, the crack increment curve appears inflection point and rises rapidly, and the number of cracks begins to grow rapidly. At this time, the filling body samples begin to enter the post-peak failure stage, and the stress–strain curve begins to decline. It can be seen from the table that with the increase in filling layers, the stress and strain when cracks appear and the crack growth rate changes gradually decrease. This shows that with the gradual increase in filling times, the filling body reaches the initial failure and peak strength faster, which further verifies the weakening effect of filling times on the strength of the filling body.

#### 3.3.3. Energy Conversion of Layered Cemented Backfill under Failure State

**Figure 9:**
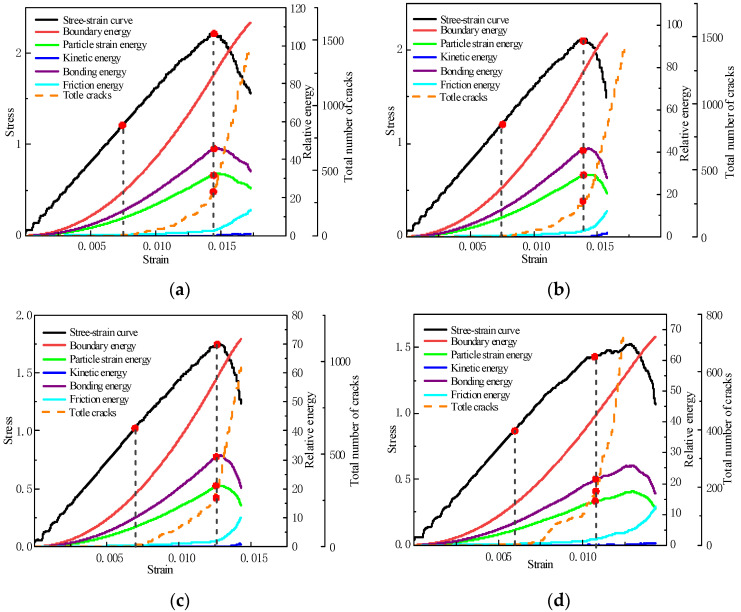
Energy conversion and stress–strain curve; (**a**) one layer, (**b**) two layers, (**c**) three layers, (**d**) four layers.

Figure 9 shows the energy conversion of horizontally layered fillings with different layers in a broken state.

According to the principle of conservation of energy, the energy is not generated by the air, nor does it disappear by the air. Before the uniaxial compression test, the internal energy of the filling sample is in a certain equilibrium state. In the compression test, with the application of external force, the original energy balance in the sample is broken, and different energies are transformed with the change in stress–strain state, which leads to cracks and final failure of the sample. Therefore, the study of the energy change in the specimen during compression plays a vital role in studying the failure mechanism of the filling body [43,44].

Through PFC2D, all kinds of energy in the process of sample compression can be accurately counted, including five kinds of energy; boundary energy, which is the energy produced by the external force on the sample; strain energy, which is the energy between particle contact; kinetic energy, which is the energy of particle motion; bond energy, which is energy stored at the bond; and friction energy, which is the friction energy between particles. In order to simplify the analysis, the deformation process of the specimen is divided into three stages: linear elastic stage (before fracture occurrence), elastic–plastic stage (from stress to peak stress at the beginning of fracture occurrence) and plastic stage (after peak stress). The strain energy stored by the sample in the linear elastic stage is all the elastic strain energy. With the gradual increase in external load, the strain energy and bond energy inside the specimen increase due to the absorption of boundary energy. When the stress of the sample reaches 55% of the peak stress, the sample enters the elastic–plastic stage. The bonding bonds between some particles cannot withstand more bond energy, and fractures and cracks begin to occur. However, due to the loss of bond bonding bonds between particles, the friction energy between particles begins to increase gradually, and the energy inside the sample continues to accumulate, accompanied by the generation of cracks. Partial elastic strain energy is converted into dissipation energy (dissipation energy = boundary energy, kinetic energy, strain energy, bond energy). When the load continues to increase, and the stress of the specimen reaches its peak stress, the specimen begins to enter the plastic stage. At this stage, the bond bonds between most particles are broken, the bond energy begins to decline, and the number of cracks begins to rise sharply. With the expansion of each old crack and the generation of new cracks, more elastic strain energy in the sample gradually transforms into dissipative energy, resulting in the gradual increase in dissipative energy. During the whole compression process, since the filling sample is solid and the particle motion is weak, the kinetic energy is small, and the changing trend is not apparent.

## 4. Conclusions

(1) The uniaxial compressive strength of layered backfill increases with the increase in slurry concentration. The increase in slurry concentration has the most obvious effect on the uniaxial compressive strength of the filling body, and the increase in the number of layers has a weakening effect on the strength of the filling body. Based on the damage theory, the constitutive damage model was constructed. It was found that the total damage of the filling body increases sharply with the increase in strain, and then increases slowly and finally gradually tends to one;
(2) By using PFC2D to analyze the fracture evolution law and failure mode of the filling body, it is found that when the stress of the filling body reaches about 55% of the peak stress, the cracks begin to appear and gradually develop; when the filling body stress reaches 90–100% of the peak stress, the number of cracks begins to rise sharply. The main failure modes of one layer and two layers backfills show obvious shear failure characteristics, and the failure modes of three layers and four layers backfills are closer to tensile failure;
(3) The generation of cracks is accompanied by the mutual transformation of different energies. In the online elastic stage, the energy in the filling body begins to accumulate, and the strain energy and bond energy increase continuously; in the elastic–plastic stage, part of the strain energy begins to transform into dissipation energy, and the friction energy gradually increases and cracks begin to produce; in the plastic stage, the strain energy continues to transform into dissipated energy, and the bond energy begins to decline. The cracks gradually develop, and the number increases sharply until the specimen is destroyed.

## Figures and Tables

**Table 1:** Chemical composition of tailings.

Class	SiO_2_	Al_2_O_3_	CaO	MgO	P	S	Fe	Au
Contents(%)	65.7	14.3	1.88	0.49	0.08	0.13	3.05	<0.01

**Table 2:** Uniaxial compressive strength of layered backfill/(MPa).

Slurry Concentration	One Filling	Two Filling	Three Filling	Four Filling
68%	1.04	0.84	0.72	0.35
70%	1.32	0.89	0.77	0.55
72%	1.82	1.65	1.36	1.26
74%	2.24	2.13	1.76	1.44

**Table 3:** Two-factor analysis of variance table.

Source	SS	DF	MS	F	P
Correction model	23.573 ^a^	15	1.572	151.731	<0.001
Intercept	126.479	1	126.479	12,211.378	<0.001
Slurry concentration	17.805	3	5.935	572.999	<0.001
Filling times	5.404	3	1.801	173.913	<0.001
Slurry concentration ∗ Filling times	0.365	9	0.041	3.914	<0.001
Error	0.663	64	0.010		
Total	150.715	80			
Revised total	24.236	79			

^a^: R^2^ = 0.973.

**Table 4:** Pairings comparison of slurry concentration.

Slurry Concentration/%	Case	Subset			
		1	2	3	4
68	20	0.7320			
70	20		0.8825		
72	20			1.5225	
74	20				1.8925
P		1.000	1.000	1.000	1.000

**Table 5:** Pairings comparison of filling times.

Filling Times	Case	Subset			
		1	2	3	4
4	20	0.9000			
3	20		1.1525		
2	20			1.3775	
1	20				1.5995
P		1.000	1.000	1.000	1.000

**Table 6:** Mechanical parameters of layered cemented filling body.

Layer Number	Peak Stress/MPa	Peak Strain/%	Elastic Modulus/GPa
1	2.24	1.45	0.154
2	2.13	1.41	0.151
3	1.76	1.27	0.139
4	1.44	1.10	0.131

**Table 7:** Parameters of damage constitutive model.

Layer Number	m	$\varepsilon_{0}/\%$
1	2.306	2.083
2	2.303	2.026
3	2.299	1.824
4	2.302	1.580

**Table 8:** The relationship between stress–strain and crack increments.

Number of Layers	Cracks Begin to Appear		Inflection Point of Crack Number Growth	
	Stress/MPa	Strain	Stress/MPa	Strain
One layer	1.23	0.0075	2.23	0.0144
Two layers	1.13	0.0069	2.10	0.0139
Three layers	0.99	0.0067	1.75	0.0127
Four layers	0.75	0.0051	1.40	0.0106

## Data Availability

Data are contained within the article.
